# Electrical storm treatment by percutaneous stellate ganglion block: the STAR study

**DOI:** 10.1093/eurheartj/ehae021

**Published:** 2024-01-30

**Authors:** Simone Savastano, Enrico Baldi, Sara Compagnoni, Roberto Rordorf, Antonio Sanzo, Francesca Romana Gentile, Veronica Dusi, Simone Frea, Carol Gravinese, Filippo Maria Cauti, Gianmarco Iannopollo, Francesco De Sensi, Edoardo Gandolfi, Laura Frigerio, Pasquale Crea, Domenico Zagari, Matteo Casula, Giuseppe Sangiorgi, Simone Persampieri, Gabriele Dell’Era, Giuseppe Patti, Claudia Colombo, Giacomo Mugnai, Francesco Notaristefano, Alberto Barengo, Roberta Falcetti, Giovanni Battista Perego, Giuseppe D’Angelo, Nikita Tanese, Alessia Currao, Vito Sgromo, Gaetano Maria De Ferrari, the STAR study group, Alessandro Fasolino, Sara Bendotti, Roberto Primi, Angelo Auricchio, Giulio Conte, Pietro Rossi, Filippo Angelini, Arianna Morena, Antonio Toscano, Valeria Carinci, Giuseppe Dattilo, Nastasia Mancini, Marco Corda, Gianfranco Tola, Giulio Binaghi, Claudia Scudu, Lucy Barone, Alessandro Lupi, Claudia Carassia, Federica De Vecchi, Sara Vargiu

**Affiliations:** Division of Cardiology, Fondazione IRCCS Policlinico San Matteo, Viale Golgi 19, 27100 Pavia, Italy; Division of Cardiology, Fondazione IRCCS Policlinico San Matteo, Viale Golgi 19, 27100 Pavia, Italy; Division of Cardiology, Fondazione IRCCS Policlinico San Matteo, Viale Golgi 19, 27100 Pavia, Italy; Department of Molecular Medicine, Section of Cardiology, University of Pavia, Pavia, Italy; Division of Cardiology, Fondazione IRCCS Policlinico San Matteo, Viale Golgi 19, 27100 Pavia, Italy; Division of Cardiology, Fondazione IRCCS Policlinico San Matteo, Viale Golgi 19, 27100 Pavia, Italy; Division of Cardiology, Fondazione IRCCS Policlinico San Matteo, Viale Golgi 19, 27100 Pavia, Italy; Department of Molecular Medicine, Section of Cardiology, University of Pavia, Pavia, Italy; Division of Cardiology, Molinette Hospital, Città della Salute e della Scienza, Torino, Italy; Department of Medical Sciences, University of Torino, Torino, Italy; Division of Cardiology, Molinette Hospital, Città della Salute e della Scienza, Torino, Italy; Department of Medical Sciences, University of Torino, Torino, Italy; Division of Cardiology, Molinette Hospital, Città della Salute e della Scienza, Torino, Italy; Department of Medical Sciences, University of Torino, Torino, Italy; Division of Cardiology, Fatebenefratelli Hospital, Rome, Italy; Division of Cardiology, Maggiore Hospital, Bologna, Italy; Division of Cardiology, Misericordia Hospital, Grosseto, Italy; Division of Cardiology, Santi Antonio e Biagio e Cesare Arrigo Hospital, Alessandria, Italy; Division of Cardiology, Santi Antonio e Biagio e Cesare Arrigo Hospital, Alessandria, Italy; Division of Cardiology, Maggiore Hospital, Crema, Italy; Division of Cardiology, G. Martino Hospital, Messina, Italy; Division of Cardiology, Humanitas Mater Domini, Castellanza, Italy; Division of Cardiology, ‘San Michele’ dell’ARNAS G. Brotzu Hospital, Cagliari, Italy; Division of Cardiology, ‘Tor Vergata’ University Hospital, Rome, Italy; Division of Cardiology, San Biagio Hospital, Domodossola, Italy; Division of Cardiology, Maggiore della carità Hospital, Novara, Italy; Division of Cardiology, Maggiore della carità Hospital, Novara, Italy; University of Eastern Piedmont ‘Amedeo Avogadro’, Novara, Italy; Division of Cardiology, ‘A. De Gasperis’, ASST Grande Ospedale Metropolitano Niguarda, Milan, Italy; Division of Cardiology, Department of Medicine, School of Medicine, University of Verona, Verona, Italy; Division of Cardiology, Santa Maria della Misericordia Hospital, Perugia, Italy; Division of Cardiology, Santa Maria della Misericordia Hospital, Perugia, Italy; Division of Cardiology, Sant’Andrea University Hospital, Rome, Italy; Istituto Auxologico Italiano – IRCCS, Milano, Italy; Department of Cardiac Electrophysiology and Arrhythmology, IRCCS San Raffaele Hospital and Vita-Salute University, Milan, Italy; Department of Cardiac Electrophysiology and Arrhythmology, IRCCS San Raffaele Hospital and Vita-Salute University, Milan, Italy; Division of Cardiology, Fondazione IRCCS Policlinico San Matteo, Viale Golgi 19, 27100 Pavia, Italy; AREU Azienda Regionale Emergenza Urgenza, AAT Pavia Fondazione IRCCS Policlinico San Matteo, Pavia, Italy; Division of Cardiology, Molinette Hospital, Città della Salute e della Scienza, Torino, Italy; Department of Medical Sciences, University of Torino, Torino, Italy

**Keywords:** Electrical storm, Neuromodulation, Stellate ganglion block, Ventricular tachycardia

## Abstract

**Background and Aims:**

An electrical storm (ES) is a clinical emergency with a paucity of established treatment options. Despite initial encouraging reports about the safety and effectiveness of percutaneous stellate ganglion block (PSGB), many questions remained unsettled and evidence from a prospective multicentre study was still lacking. For these purposes, the STAR study was designed.

**Methods:**

This is a multicentre observational study enrolling patients suffering from an ES refractory to standard treatment from 1 July 2017 to 30 June 2023. The primary outcome was the reduction of treated arrhythmic events by at least 50% comparing the 12 h following PSGB with the 12 h before the procedure. STAR operators were specifically trained to both the anterior anatomical and the lateral ultrasound-guided approach.

**Results:**

A total of 131 patients from 19 centres were enrolled and underwent 184 PSGBs. Patients were mainly male (83.2%) with a median age of 68 (63.8–69.2) years and a depressed left ventricular ejection fraction (25.0 ± 12.3%). The primary outcome was reached in 92% of patients, and the median reduction of arrhythmic episodes between 12 h before and after PSGB was 100% (interquartile range −100% to −92.3%). Arrhythmic episodes requiring treatment were significantly reduced comparing 12 h before the first PSGB with 12 h after the last procedure [six (3–15.8) vs. 0 (0–1), *P* < .0001] and comparing 1 h before with 1 h after each procedure [2 (0–6) vs. 0 (0–0), *P* < .001]. One major complication occurred (0.5%).

**Conclusions:**

The findings of this large, prospective, multicentre study provide evidence in favour of the effectiveness and safety of PSGB for the treatment of refractory ES.

## Structured Graphical Abstract

**Structured Graphical Abstract:**
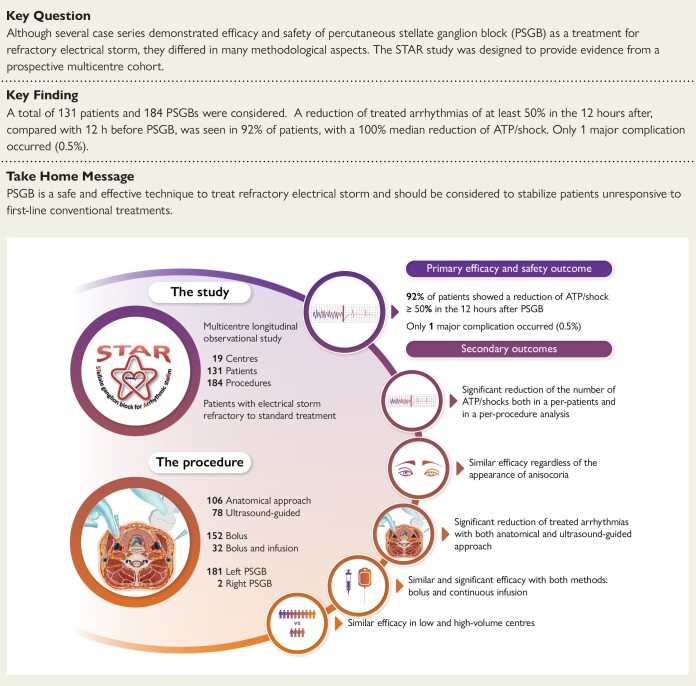
Methods and main findings of the STAR study. ATP, antitachycardia pacing; PSGB, percutaneous stellate ganglion block.

**See the editorial comment for this article ‘Stellate ganglion blockade for the management of ventricular arrhythmia storm', by V. Malik and K. Shivkumar, https://doi.org/10.1093/eurheartj/ehae083.**

## Introduction

The management of an electrical storm (ES), classically defined as the occurrence of three or more episodes of ventricular tachycardia (VT) or ventricular fibrillation (VF) in 24 h,^1,2^ is extremely challenging, particularly in the case of refractory cardiac arrest or of rapidly recurrent ventricular arrhythmias. ES is a relatively common condition, occurring in ∼5% of patients with an implantable cardioverter defibrillator (ICD) implanted for primary prevention and in 25% of patients implanted for secondary prevention.^3,4^ Traditional treatments, either pharmacological or non-pharmacological,^5,6^ are few and often not rapidly effective or available at all hospitals. Therefore, there is an unmet need for additional therapeutic options. In this setting, percutaneous stellate ganglion block (PSGB) may provide a significant contribution to approach this life-threatening condition. The close relationship between sympathetic activation and ventricular arrhythmias was elegantly demonstrated back in the 1970s^7,8^ and, during that era, PSGB was first used on a patient for antiarrhythmic purposes.^9^ Thereafter, several isolated cases were reported and collected in the meta-analysis by Fudim in 2017^10^ which suggested a significant reduction of arrhythmic events after PSGB. Subsequently, six case series have been published,^11–16^ of which the largest enrolled 30 patients.^14^ Although they all agreed on a marked efficacy and safety of PSGB,^17^ they differed in many methodological aspects.^17^ Techniques differed concerning both approach (i.e. anatomical landmark or ultrasound-guided) and type of anaesthetic administration (i.e. single bolus, repeated bolus, or continuous infusion). Different time frames before and after PSGB were considered, and different analyses were performed (i.e. per-procedure analysis and per-patient analysis). Moreover, these small case series, which probably include procedures performed only by a few operators, could not assess the generalizability of the approach. We designed the STAR study to try to overcome all these limitations and to clearly assess the effectiveness and safety of PSGB on a larger sample from multiple centres.

## Methods

### Type of study

The STAR study (STellate ganglion block for Arrhythmic stoRm) is a multicentre international retrospective and prospective observational longitudinal study coordinated by the Fondazione IRCCS Policlinico San Matteo in Pavia and approved by the Ethical Committee of the Fondazione IRCCS Policlinico San Matteo (proc. 20190046932) and by the competent Committees for all the participating centres. The list of the participating centres together with the corresponding number of patients enrolled and of procedures provided is presented in Supplementary data online, *Figure S1*. The STAR study was regularly registered on ClinicalTrials.gov (NCT05720936) and follows the Strengthening the Reporting of Observational Studies in Epidemiology (STROBE) reporting guideline (see Supplementary data online, *Appendix S1*).

### Definitions

ES was defined as the presence of three or more separate episodes of VT or VF (either sustained or requiring treatment) within 24 h.^1,2^ ES was defined as refractory in the case of arrhythmic relapses despite i.v. administration of antiarrhythmic drugs (AADs). The patients who required discontinuation of AAD i.v. infusion due to an adverse event were also considered eligible for the study.

An arrhythmic event was defined as an episode of sustained VT or VF treated either with antitachycardia pacing (ATP) or with a direct current (DC) shock by an internal or an external defibrillator. Sustained ventricular arrhythmias that self-terminated without ATP and/or shock, as well as non-sustained ventricular arrhythmias, were not considered.

Pre-specified complications included: simple haematoma, haematoma requiring intervention, symptoms due to anaesthetic absorption, intravascular injection with or without complication, brachial plexus damage, simple vascular damage, and vascular damage requiring intervention. Additionally, investigators were required to report any other kind of procedure-related potential side effects they observed. Centres who enrolled ≥20 patients over the study period were defined as ‘high volume’.

### Study outcomes

The primary outcome was the effectiveness of the PSGB defined as a reduction of arrhythmic events (number of ATPs or DC shocks) of at least 50% in the 12 h immediately after the block compared with the 12 h immediately before.

Secondary outcomes were: (2.1) the comparison of the number of ATPs/DC shocks in the 12 h immediately after PSGB as compared with the 12 h immediately before; (2.2) the feasibility of the procedure expressed as the number of complications (see definitions) within 12 h from the procedure; (2.3) the comparison of the effectiveness of PSGB in patients who developed anisocoria after PSGB and those who did not; (2.4) the comparison of the effectiveness of PSGB in patients who were treated with the anterior anatomical approach and those treated with the lateral ultrasound-guided approach; (2.5) the comparison of the effectiveness of PSGB in patients who received only single-shot PSGB and in patients who also received continuous PSGB; and (2.6) the comparison of the effectiveness of PSGB in high- vs. low-volume centres.

### Patient selection

We enrolled all consecutive patients who underwent PSGB for the treatment of refractory ES according to the study protocol in one of the participating centres from 1 July 2017 to 30 June 2023. Patients younger than 18 years, patients with a history of heart transplant or previous surgical cardiac sympathetic denervation, and patients whose neck was judged by the operator as not suitable for the procedure (previous neck surgery, previous burns, presence of large scars, or thyroid goitre) were excluded.

### Electrical storm management

ES was managed according to standard clinical practice at each institution. Possible treatments included resolution of reversible causes (e.g. electrolyte imbalances, acute myocardial ischaemia, septic status, or cardiogenic shock), administration of drugs such as AADs and beta-blockers, ICD reprogramming to minimize shocks, possible use of mechanical circulatory support devices (e.g. intra-aortic balloon pump or extracorporeal membrane oxygenation), and general anaesthesia. Each centre was free to consider PSGB at any time as long as ES was refractory to the ongoing treatments.

### Percutaneous left stellate ganglion block procedure

**Figure 1:**
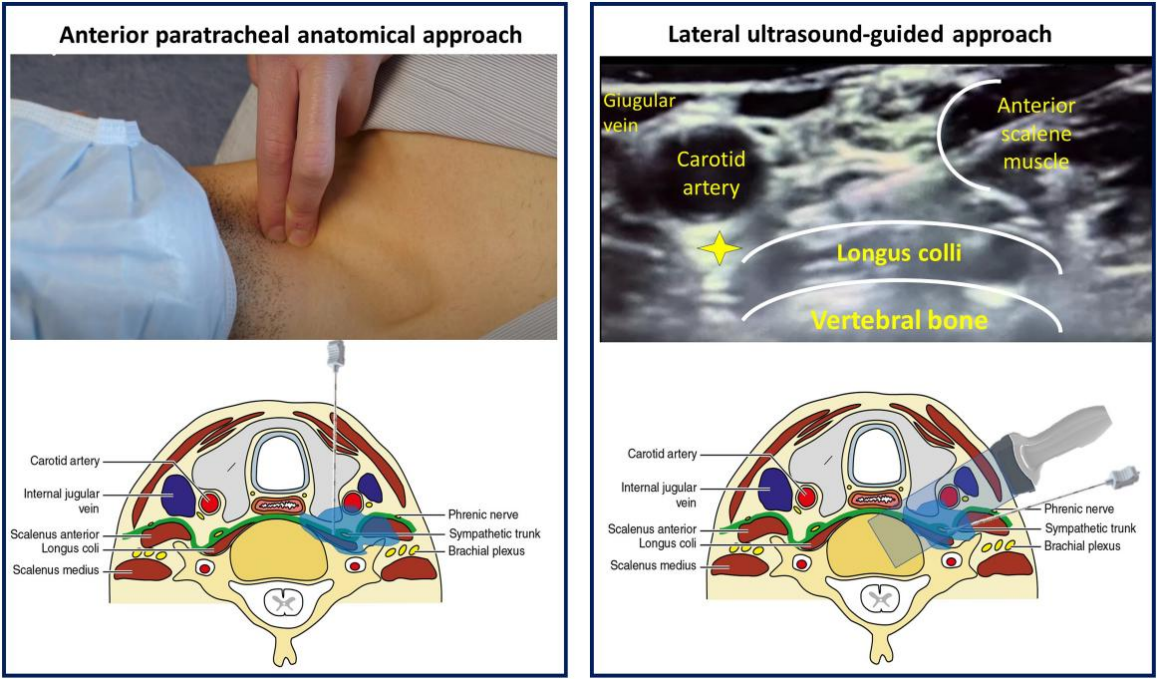
Schematic depiction of the two approaches. On the left, the anterior paratracheal anatomical approach is shown. In the higher part of the left panel, the position of the hand and the site of puncture are illustrated. In the right panel, the later ultrasound-guided approach is shown. In the upper part of the right panel, the ultrasound landmarks are represented, and the yellow star indicates the target zone

The technique of PSGB (anterior anatomical or lateral ultrasound-guided approach) was left to the operators according to their expertise and preference, provided that they followed the STAR standards (see operators’ training). They were also free to shift from one approach to the other for the same patient in the case of repeated procedures. The anatomical approach consisted of a paratracheal anterior injection of a short- and/or long-acting anaesthetic at the level of the left-sided Chassaignac’s tubercle (C6).^18^ The needle was advanced perpendicularly to the skin up to the bone of the transverse process of C6 and then minimally retracted before injection. The ultrasound-guided lateral technique consisted of an intrascalenic approach with the injection of a short- and/or a long-acting anaesthetic over the longus colli muscle and below the carotid artery (*Figure 1*). The manoeuvres were identical regardless of the side of injection.

The choice of the type of anaesthetic as well as the decision to perform a single-shot block, to repeat the single-shot block, and/or to leave a catheter in place for continuous infusion (continuous PSGB) was left to the operator and the physician in charge. Repetition of PSGB was considered in the case of arrhythmic recurrence within or after the period of effectiveness of the anaesthetic used. In the case of early recurrences after the period of anaesthetic effectiveness or in the case of medical conditions triggering the ES and expected to require a relatively long time for their resolution (e.g. septic shock or thyrotoxicosis), a continuous infusion might have been considered directly after the first bolus. Regardless of the approach, an aspiration check was performed before the injection. Moreover, considering the emergency setting, the procedure was performed regardless of any anticoagulant or antiplatelet therapy. For continuous infusion, a spring-wound epidural catheter was left in place for anaesthetic infusion.

Concerning the side of the block, the first two attempts were performed on the left side. The right side could be considered in the case of further recurrences within the presumed time frame of the effect of the anaesthetic used.

### Operators’ training

**Figure 2:**
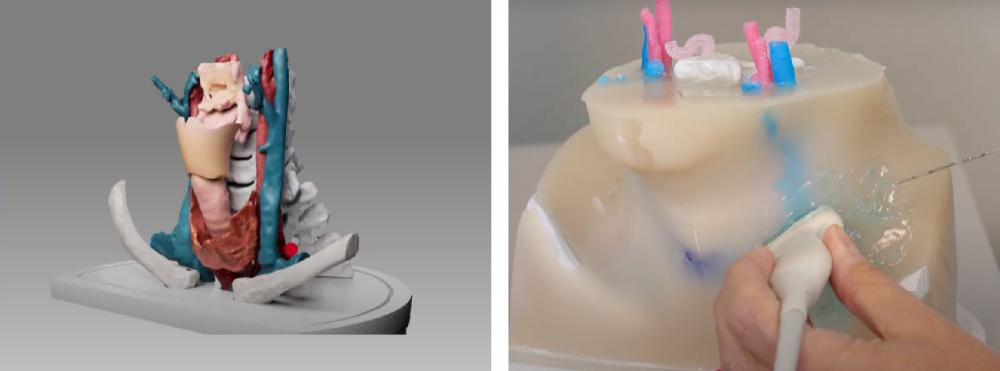
The ad-hoc 3D model designed for operators’ training. This model consists of 3D printed anatomical structures (on the left) plunged in an agar-based gel (on the right) which produces the same echogenicity as the human body

All the operators were trained with a specifically designed 8-h course planned and promoted by the Fondazione IRCCS Policlinico San Matteo in Pavia. Our training courses were aimed at clinical cardiologists, interventional cardiologists, intensivists, and emergency physicians. The course consisted of 4 h of theory lessons followed by 4 h of practical training (see Supplementary data online, *Table S1*). Trainees learned both the anterior anatomical and the lateral ultrasound-guided approach. All the operators were trained to identify both the anatomical (i.e. the Chassaignac’s tubercle) and the ultrasound landmarks on at least two different healthy volunteers and to perform PSGB on a special ad-hoc made dummy neck. This included the use of a patient-specific 3D printed model which offers the possibility of fidelity training (*Figure 2*). During the training course, the management of possible complications was discussed. After passing the final multiple-choice test, participants were considered STAR providers. Every centre was required to have at least one trained investigator and to have received ethical approval before enrolling patients.

### Data collection

The data of the study are collected and managed using REDCap®^19,20^ electronic data capture tools hosted at Fondazione IRCCS Policlinico San Matteo. The electronic case report form (eCRF) consisted of three parts focused on patient characteristics, PSGB details, and outcomes (the eCRF is available in Supplementary data online, *Appendix S2*). Considering that more than one PSGB was allowed for each patient, this electronic sheet could be duplicated for each PSGB performed.

### Sample size

We selected our sample size to have a lower boundary of the 95% confidence interval (CI) for efficacy (i.e. ≥50% reduction in treated ventricular arrhythmias) > 70%, considered the least clinically meaningful result. Based on our previous experience, represented by the retrospective cohort of patients, the efficacy of PSGB was estimated to be 90%. One hundred patients providing reliable data for the primary outcome analysis allowed 94% power for the lower boundary of the efficacy CI to exceed 70% with an alpha two-sided error of 5%.

### Statistical analysis

Categorical variables are presented as numbers and percentages, and were compared with a χ^2^ test. Continuous variables were tested for normality with the D’Agostino–Pearson test. Normally distributed continuous variables are presented as the mean ± standard deviation (SD) and compared with Student’s *t*-test. Non-normally distributed continuous variables are presented as the median and 25%–75% interquartile range (IQR) and were compared with the Mann–Whitney *U* test if independent or with the Wilcoxon test in the case of paired variables.

For the analysis of the primary outcome, the reduction of treated arrhythmic episodes was computed and expressed as a percentage. Concerning the analysis of the secondary outcomes, the number of ATPs/DC shocks before and after the PSGB was compared with the Wilcoxon matched paired sign rank test (outcome 2.1); the number of complications was computed and presented as count and percentage (outcome 2.2). Concerning outcomes from 2.3 to 2.6, the effectiveness in the pre-defined subgroups was managed as for outcome 2.1 and the resulting Hodges–Lehmann median differences, together with their 95% CI, between groups were compared.

A *P*-value <.05 was considered statistically significant. Analyses were performed using a statistical software (MedCal software version 12.5.0.0 by MedCal software Ltd). Patients with missing data were not considered for analysis.

## Results

### Patient and procedure characteristics

**Figure 3:**
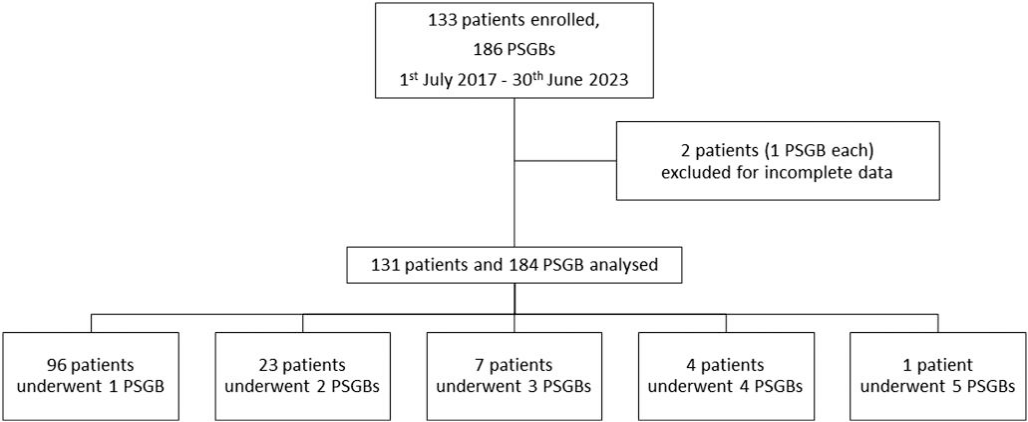
Study flow diagram

Over the study period, 133 patients were enrolled from 19 centres, and 2 patients with incomplete data were excluded, resulting in a final cohort of 131 patients (*Figure 3*). They were mainly males (83.2%) and the median age was 68 (63.8–69.2) years. Most patients had a structural heart disease (including 29% with acute myocardial infarction) and the mean left ventricular ejection fraction (LVEF) was markedly depressed (25.0 ± 12.3%); a quarter were in cardiogenic (21%) or septic (5%) shock and 11 patients (8%) were in cardiac arrest. Regarding chronic oral medications, 63% of patients were on beta-blocker treatment, 35% were taking amiodarone, 5% sotalol, and 16% mexiletine (*Table 1*).

**Table 1:** Patients’ baseline characteristics (*n* = 131)

Characteristic	*n* = 131
Age, years^a^	68 (57–76)
Male sex, *n* (%)^b^	109 (83.2)
LVEF, %^c^	25.0 ± 12.3
**Causative diagnosis of electrical storm, *n* (%)^b^**	
ST-elevation myocardial infarction	23 (17.6)
Non-ST-elevation myocardial infarction	14 (10.7)
Chronic coronary artery disease	37 (28.2)
Dilated cardiomyopathy	29 (22.1)
Arrhythmogenic cardiomyopathy	4 (3.1)
Hypertrophic cardiomyopathy	2 (1.5)
Myocarditis	4 (3.1)
Valvular heart disease	2 (1.5)
Long QT syndrome	1 (0.8)
Hypokalaemia	2 (1.5)
Acute pulmonary oedema	1 (0.8)
Heart failure in LVAD bearer	1 (0.8)
Idiopathic short-coupled Torsades de Pointes	1 (0.8)
Idiopathic ventricular fibrillation	1 (0.8)
Methadone overdose	1 (0.8)
Takotsubo syndrome with long QT interval	1 (0.8)
Sepsis	3 (2.3)
Sepsis in COVID-19 infection	1 (0.8)
Septic shock	1 (0.8)
Pneumonia	1 (0.8)
Post-chemotherapic cytokine release syndrome	1 (0.8)
Refractory cardiac arrest, *n* (%)^b^	11 (8.4)
Cardiogenic shock, *n* (%)^b^	27 (20.6)
Septic shock, *n* (%)^b^	7 (5.3)
Diabetes, *n* (%)^b^	41 (31.3)
**Oral drug at admission, *n* (%)^b^**	
Beta-blockers	82 (62.6)
Amiodarone	46 (35)
Sotalol	6 (5)
Mexiletine	21 (16)
In-hospital death, *n* (%)^b^	36 (27.5)
Electrical storm	6 (4.6)
Cardiogenic shock	16 (12.2)
Septic shock	8 (6.1)
Sepsis	2 (1.5)
Acute respiratory failure	1 (0.8)
Pulmonary embolism	1 (0.8)
Pneumonia	1 (0.8)
Multiorgan failure after LVAD placement	1 (0.8)
VT ablation after PSGB, *n* (%)^b^	34 (26)
Cardiac sympathetic denervation after PSGB, *n* (%)^b^	12 (9.2)
ATPs/shocks in the 12 h before the first PSGB^a^	6 (3–15.8)
ATPs/shocks in the 12 h after the last PSGB^a^	0 (0–1)

LVAD, left ventricular assist device; LVEF, left ventricular ejection fraction; VT, ventricular tachycardia; ATP, antitachycardia pacing; PSGB, percutaneous stellate ganglion block.

^a^Median (IQR).

^b^Number (proportion).

^c^Mean ± standard deviation.

A total of 184 PSGBs were performed, with either an anatomical (57.6%) or an ultrasound-guided approach (42.4%). Considering all the procedures, bolus administration and bolus followed by continuous infusion accounted for 82.6% and 17.4% of cases, respectively. One-fifth of the procedures were performed in intubated patients with only 14% in patients taking neither anticoagulant nor antiplatelet therapy. Concerning i.v. AAD before each procedure, most PSGBs (88%) were performed on patients receiving lidocaine or amiodarone, either alone or in combination (33%); 3% were performed on patients receiving procainamide. Most of the procedures (64%) were provided for VT as causative arrhythmias of the ES, with 17% for both VT and VF. Three procedures were performed at the right side for early arrhythmic relapses during the time frame of anaesthetic action in three different patients suffering from ES complicating an ischaemic dilated cardiomyopathy. *Table 2* shows in detail the procedure characteristics.

**Table 2:** Characteristics of PSGBs (*n* = 184)

Characteristic	*n* = 184
**Approach, *n* (%)^a^**	
Anterior anatomical	106 (57.6)
Lateral ultrasound-guided	78 (42.4)
**Side, *n* (%)^a^**	
Left	181 (98.4)
Right	3 (1.6)
**Mode of administration, *n* (%)^a^**	
Bolus	152 (82.6)
Bolus and infusion	32 (17.4)
**Anaesthetic used for bolus, *n* (%)^a^**	
Lidocaine	53 (28.8)
Bupivacaine	8 (4.3)
Ropivacaine	1 (0.5)
Mepivacaine	3 (1.7)
Lidocaine + bupivacaine	44 (23.9)
Lidocaine + ropivacaine	63 (34.2)
Lidocaine + mepivacaine	11 (6)
Lidocaine + levobupivacaine	1 (0.5)
**Anaesthetic used for infusion, *n* (%)^a^**	
Lidocaine	21 (65.6)
Ropivacaine	11 (34.4)
**Anaesthetic infusion duration, min^b^**	3660 (1440–7203.8)
**Type of arrhythmias during electrical storm, *n* (%)^a^**	
VT	118 (64.1)
VF	35 (19)
VT and VF	31 (16.8)
**VT cycle length, ms^b^**	360 (340–378.7)
**Pre-PSGB intervention, *n* (%)^a^**	
Intubation	37 (20.1)
Sedation	40 (21.7)
IABP	18 (9.8)
ECMO	7 (3.8)
**Anticoagulant/antiplatelet therapy, *n* (%)^a^**	
None	26 (14.1)
SAPT only	35 (19)
SAPT + heparin	29 (15.8)
SAPT + VKA/DOAC	6 (3.3)
DAPT only	13 (7.1)
DAPT + heparin	16 (8.7)
DAPT + VKA/DOAC	1 (0.5)
Heparin only	24 (13)
VKA/DOAC only	34 (18.5)
**Pre-PSGB refractory cardiac arrest, *n* (%)^a^**	14 (7.6)
**Pre-PSGB i.v. medication, *n* (%)^a^**	
Amines	47 (25.5)
Amiodarone	34 (18.5)
Lidocaine	67 (36.4)
Amiodarone and lidocaine	61 (33.2)
Procainamide	5 (2.7)
Beta-blockers	21 (11.4)
**Post-PSGB anisocoria, *n* (%)^a^**	
Yes	72 (39.1)
No	103 (56)
Unknown	9 (4.9)
**PSGB major complications, *n* (%)^a^**	
Respiratory depression	1 (0.5)
**PSGB minor complications, *n* (%)^a^**	
Bradycardia	1 (0.5)
Hypotension	1 (0.5)
**PSGB described side effects, *n* (%)^a^**	
Temporary brachial plexus paralysis	3 (1.6)
Hoarseness	2 (1.1)
Dysphonia	1 (0.5)
Neck pain	1 (0.5)
Vomiting	1 (0.5)

VT, ventricular tachycardia; VF, ventricular fibrillation; PSGB, percutaneous stellate ganglion block; IABP, intra-aortic balloon pump; ECMO, extracorporeal membrane oxygenation; SAPT, single antiplatelet therapy; VKA, vitamin K antagonist; DOAC, direct oral anticoagulant; DAPT, dual antiplatelet therapy.

^a^Number (proportion).

^b^Median (IQR).

### Primary outcome

Most of the 131 patients enrolled (115, 88%) suffered arrhythmic episodes requiring treatment in the 12 h before PSGB. Among them, 106 (92.2%) showed a reduction in the number of treated episodes of ≥50% in the 12 h after the procedures (median reduction 100%, IQR −100% to −92.3%).

### Secondary outcomes

**Figure 4:**
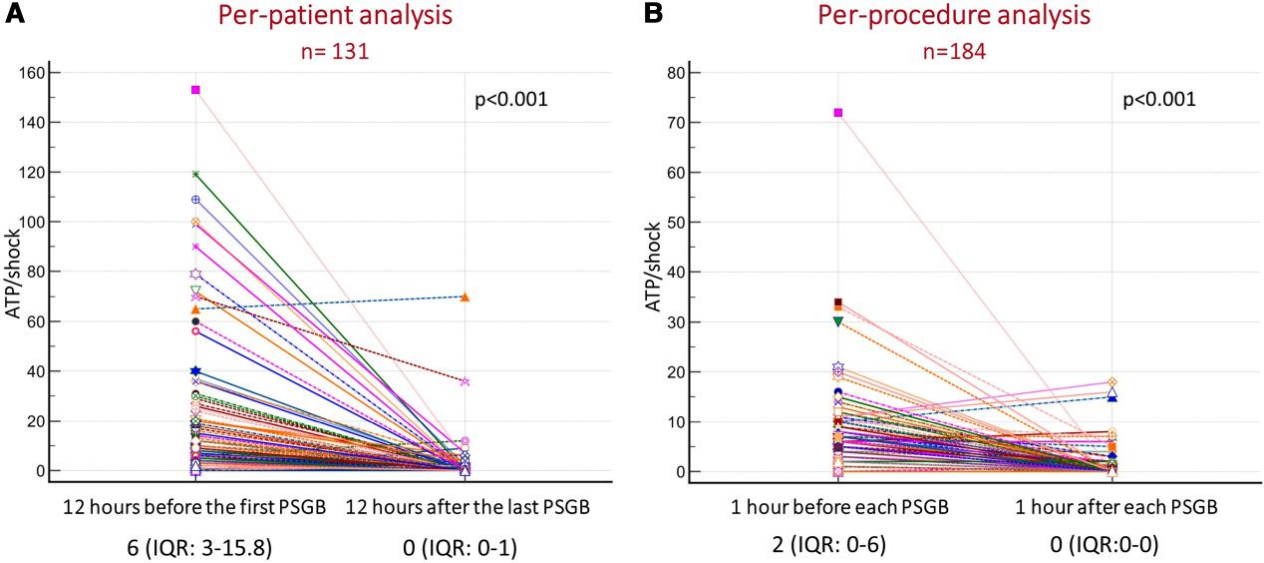
The per-patient analysis (*A*) and the per-procedure analysis (*B*) comparing the number of arrhythmic episodes before and after PSGB. Wilcoxon matched paired signed rank test with resulting *P*-value is displayed

For the secondary outcomes, 131 patients (per-patient analysis) and 184 procedures (per-procedure analysis) were considered. In the per-patient analysis, the median number of treated episodes in the 12 h after the last procedure was significantly lower compared with the 12 h before the first procedure [0 (IQR 0–1) vs. 6 (IQR 3–15.8), *P* < .001] (*Figure 4A*). Supplementary data online, *Figure S2* shows the arrhythmic burden for each patient in the 12 h before the first PSGB and in the 12 h after the last PSGB.

In the per-procedure analysis, the number of treated arrhythmic episodes in the first hour after every procedure was significantly lower compared with the hour immediately preceding each procedure [0 (IQR 0–0) vs. 2 (IQR 0–6), *P* < .001] (*Figure 4B*). The reduction in the number of treated arrhythmias was similar and statistically significant after the first, the second, and further PSGBs, and the absolute number of ATPs or DC shocks was similar before the first, the second, or further PSGBs (see Supplementary data online, *Table S2*).

Regarding safety, we observed only one (0.5%) major complication, most probably due to local anaesthetic systemic toxicity (LAST). A patient on high-dose i.v. lidocaine suffered respiratory depression. The patient was treated with lipid infusion according to the provided recommendations, with no further complications. Self-terminating side effects and two minor complications are listed in *Table 2*.

**Figure 5:**
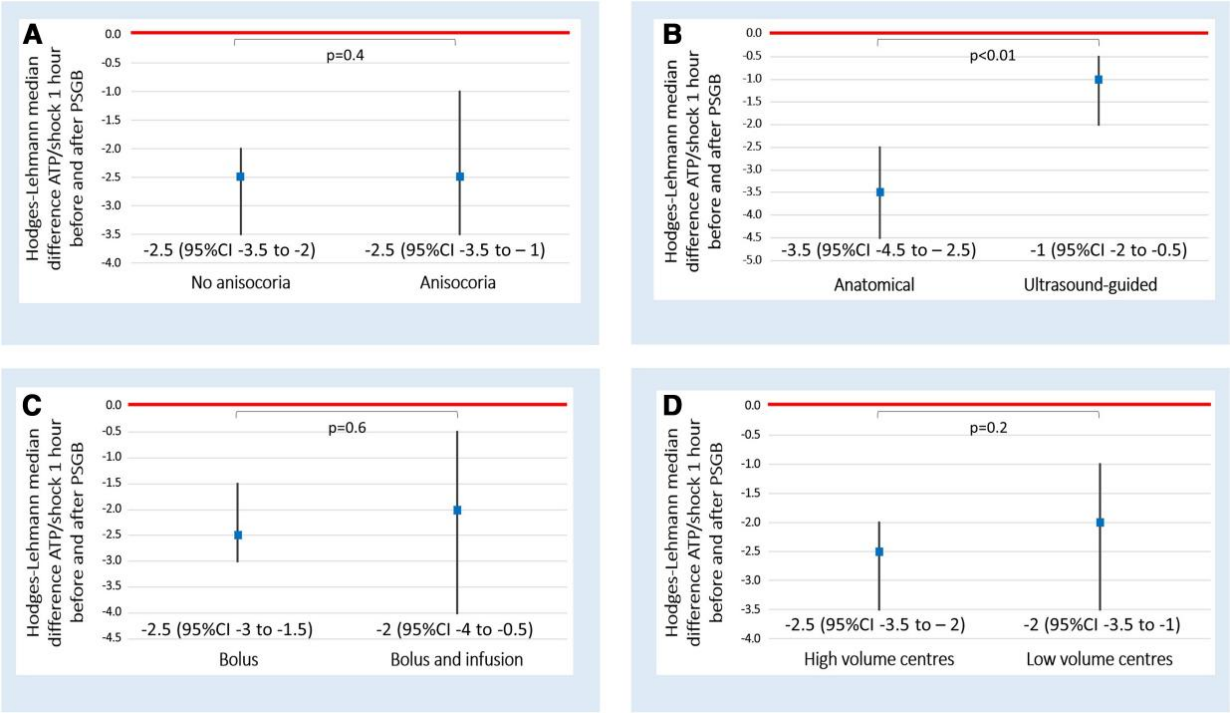
Subgroup per-procedure analysis providing the comparison of Hodges–Lehmann median differences of the number of ATPs/shocks 1 h before and after PSGB between the different subgroups. Panel (*A*): anisocoaria vs no anisocora; panel (*B*): anatomical vs ultrasound-guided approach; panel (*C*): bolus vs bolus and infusion and panel (*D*): high volume vs low volume centres

In the per-procedure analysis, we showed a similar significant reduction of treated arrhythmic episodes in patients who developed anisocoria and in those who did not [−2.5 (95% CI −3.5 to −1) vs. −2.5 (95% CI −3.5 to −2), *P* = .4] (*Figure 5A*).

The comparison of the two approaches (anatomical landmark and ultrasound-guided) in a per-procedure analysis revealed a statistically significant reduction of the arrhythmic episodes in both groups, with a decrease that was significantly higher in those managed by the anatomical-based approach [−3.5 (95% CI −4.5 to −2.5) vs. −1 (95% CI −2 to −0.5), *P* < .01] (*Figure 5B*). Notably, patient characteristics were different when comparing the two approaches. In the anatomical group, the number of treated arrhythmias in the hour before the procedures was higher [4 (IQR 4–8.2) vs. 0 (IQR 0–3), *P* < .01], the causative arrhythmia was more frequently VF (28% vs. 7%, *P* < .001), in the case of VT the cycle length was shorter [337 (300–390) ms vs. 389 (308–429) ms, *P* = .029], and the procedure was performed more frequently during cardiac arrest (13% vs. 0%, *P* < .001) (see Supplementary data online, *Table S3*).

Comparing the number of treated arrhythmic episodes 1 h before and after each bolus with or without a subsequent continuous infusion, we found a statistically significant reduction of arrhythmic episodes overall, without significant differences between groups [−2.5 (95% CI −3 to −1.5) vs. −2 (95% CI −4 to −0.5), *P* = .6] (*Figure 5C*). The time to the first treated arrhythmia recurrence after the procedure was significantly longer in patients treated with bolus and continuous infusion compared with those treated with single bolus [360 (IQR 153–1351) min vs. 105 (IQR 5–420) min, *P* = .019]. In particular, 13 (40%) out of 32 continuous infusions were performed on patients with fever or septic/cardiogenic shock or thyrotoxicosis. A continuous infusion was considered as the first attempt in 14/32 (44%), as the second in 10/32 (31%), and as the third in 8/32 (25%). When used, continuous infusion was performed with lidocaine (21/32, 66%), with a median infusion rate of 1.66 (1.6–1.7) mg/min, or with ropivacaine (11/32, 34%), with a median infusion rate of 0.2 (0.1–0.2) mg/min.

Finally, we ran the per-procedure analysis comparing high-volume with low-volume enrolment centres and found that the reduction of treated arrhythmic episodes was statistically significant in both types of centres and the decrease was similar in the two groups [−2.5 (95% CI −3.5 to −2) vs. −2 (95% CI −3.5 to −1), *P* = .2] (*Figure 5D*).

## Discussion

This multicentre prospective study, which represents the largest experience ever reported on PSGB, provides important new evidence on the efficacy of PSGB in patients suffering from an ES and significantly increases our understanding with regard to the characteristics of this approach.

The strong connection between the autonomic nervous system and ventricular arrhythmias was elegantly demonstrated in the 1970s^7,8^ and recently reviewed.^5,17,21^ Neuronal sympathetic activation decreases the VF threshold and ventricular refractoriness.^7,8^ Neuronal sympathetic activation increases spatial dispersion of repolarization and, in the setting of structurally abnormal hearts, even paradoxically prolongs activation time,^22^ making it more likely for all types of ventricular arrhythmias to occur and perpetuate. It is now well established that the cardiac autonomic nervous system undergoes a profound remodelling in the setting of structural heart disorders, leading to a chronically increased sympathetic output combined with a decrease in vagal activity.^23^ In addition, a vicious and self-perpetuating circle begins during ES. Each arrhythmic episode, depending on the cycle length and the underlying cardiac function, may cause a variable degree of reduction in both cardiac output and blood pressure. The consequent baroreflex activation further activates sympathetic reflexes, resulting in a massive myocardial release of norepinephrine that increases the likelihood of arrhythmic recurrence. This circle may also be supported by the positive feedback spinal cardiac sympathetic afferent reflexes activated by an increased afferent nerve activity, resulting in enhanced and continuous activation of the stellate ganglion.^24^ With PSGB, we can effectively break this vicious circle and restore a stable rhythm that may last beyond the anaesthetic duration effect. However, clinical data on the efficacy of PSGB consist of isolated case reports and rather small case series. In 2017, two reviews were published by Fudim^10^ and Meng,^25^ collecting 35 and 38 patients, respectively, and showing a strong reduction in the arrhythmic burden. Over the following years, six more recent case series^11–16^ were published for an overall total of 103 patients treated with PSGB that again confirmed its antiarrhythmic efficacy.

Even though a randomized study is needed to draw final conclusions, the present study provides evidence of the effectiveness and safety of PSGB derived from a population of patients with ES which is larger than the sum of all patients previously reported. Moreover, our study includes patients from 19 different centres, overcoming the limitation of single-centre studies, as suggested by a recent document of the European Heart Rhythm Association on this topic.^6^ More than 92% of patients reached the primary outcome (reduction of at least 50% of the arrhythmic episodes after PSGB) (*Structured Graphical Abstract*) and, remarkably, in three-quarters of our patients the arrhythmic burden decreased by 93% or more. Both the extent of the reduction in arrhythmias and the rapidity of onset were remarkable. The per-procedure analysis showed that in the very first hour after the procedure, three-quarters of patients had no recurrences. Our results delineate PSGB as a highly effective technique reinforcing the role of neuromodulation in the management of ES. The most recent European guidelines for the treatment of ventricular arrhythmias^26^ have introduced the use of neuromodulation, but only in limited cases of ES and with a low level of recommendation. The present results further reinforce our previous suggestion^17^ to widen the current indications, especially when taking into account the very low rate of complications. In the present study, only one major complication (0.5%) occurred, which was due to systemic lidocaine absorption. Notably, the patient was also receiving an i.v. lidocaine infusion and the complication rapidly resolved with lipid infusion. These safety data are in agreement with the report by Moore, in the 1950s,^18^ of no serious complications in ∼2000 PSGBs performed for antalgic purposes using the anatomical technique, the same as we used for most of the patients in the present study. Similar results were shown in a more recent review which also considered imaging-guided techniques.^27^ Safety data are remarkable in that 67% of the procedures were performed in patients on dual antiplatelet therapy and/or anticoagulant therapies which are two major contraindications for thoracic epidural anaesthesia (TEA),^28^ which is a guideline-recommended alternative to PSGB for the management of ES.^26^ This consideration makes PSBG more appropriate compared with TEA for emergent conditions in patients on antiplatelet or anticoagulant agents.

Determination of mortality was not a goal of the present study; however, we observed a 27% all-cause mortality and a 5% ES-related mortality which compares favourably with reported cohorts of patients with ES.^1^ This appears promising taking into account the markedly depressed LVEF of our cohort and the knowledge that an LVEF <35% increases all-cause mortality 10-fold in patients with ES.^29^ Moreover, a quarter of the patients were in shock (21% cardiogenic and 5% septic) and 11 (8%) were in cardiac arrest, conditions notoriously associated with an extremely high risk of in-hospital death.

The present study also sheds light on some still open questions and helped overcome several, albeit not all, limitations of previous studies. The first regards the clinical usefulness of the appearance of anisocoria, which has often been considered as a marker of an effective cardiac neuronal block. We showed that the development of anisocoria was not related to the antiarrhythmic effect at the cardiac level. In fact, both in patients who did and in those who did not develop anisocoria, the arrhythmic burden was significantly reduced after PSGB, and the size of the effect was similar. The main reason for this could be related to the site of anaesthetic effect: it could block only cardiac fibres and not ocular fibres or it could block both ocular and cardiac fibres. Another possible explanation may be related to the anaesthetic distribution pathways. Hogan^30^ and colleagues described how contralateral spreading of anaesthetic could be possible after PSGB. So, it is also possible that anisocoria in some cases was concealed by an unwanted bilateral effect on ocular fibres.

The second topic concerns the technique used to approach the stellate ganglion. The case series published so far are inhomogeneous regarding the approach used for PSGB. One used only the anatomical landmark approach^11^ while the others used an ultrasound-guided approach.^12–14,16^ However, none of these provided a comparison between the two approaches. STAR operators were trained in both techniques and left free to decide which one was the most suitable for every specific situation. The present study suggests that PSGB performed with either approach significantly reduces the number of treated arrhythmic episodes. The apparent statistical superiority of the anatomical approach is probably due both to the higher number of arrhythmias treated in the hour before PSGB and to the higher number of procedures performed in the anatomical group. It is not surprising that a faster approach, such as the anatomical one, has been used in patients with a higher arrhythmic burden, and this is in line with our suggestion. This result suggests the usefulness of being capable of performing PSGB by both approaches in order to choose the best one given the clinical scenario (e.g. cardiac arrest, out-of-hospital setting, and unavailability of ultrasound).

The present study suggests similar efficacy for bolus and continuous infusion of anaesthetic at the level of the stellate ganglion, albeit the time free from arrhythmia was longer after a continuous infusion. It is plausible that a continuous infusion allows more time to resolve any potentially reversible causes precipitating an ES. Moreover, continuous infusion may also be associated with higher plasma concentrations of the anaesthetic due to both increased time of exposure and volume of anaesthetic resulting in increased systemic absorption.^31,32^

Finally, we compared high- vs. low-enrolment volume centres and we found that the procedure was significantly effective in both types of centres. This finding is a non-negligible result since it means that relatively simple training may be sufficient to perform PSGB in a safe and effective way from the very first procedure and therefore that this approach may be generalizable and up-scaled.

Another element worth discussing is the timing of PSGB relative to intubation. In the algorithms for ES management proposed by Bradfield in 2018,^33^ by Kowlgi in 2020,^2^ and in the latest ESC guidelines,^26^ neuromodulation is recommended after intubation. General anaesthesia may play a role in arrhythmias because it can decrease sympathetic activity. Differences between various anaesthetics have been shown,^34^ and several reports have suggested a potentially beneficial effect of propofol in particular.^35–38^ However, intubation is associated with potential risk^39^ that appears greater than those associated with PSGB. In the present study, 20% of PSGB procedures were performed on intubated patients. Two potential conclusions can be drawn from these data. The first is that despite intubation and general anaesthesia, arrhythmias may recur in a non-negligible number of cases. The second is that 80% of the procedures were performed on awake patients, indicating that PSGB possibly may prevent some patients from being intubated and protected from the associated risk.

### Limitations

The present study suffers from some limitations. First, this is an observational study lacking a control group. The large sample and the magnitude of the observed effect appear nonetheless sufficient to draw a favourable conclusion on the risk–benefit ratio of PSGB. Second, the STAR operators were free to decide when to provide PSGB as long as the patients had an ES. Following the definition of ES and of an arrhythmic event, some patients were expected to be free from events in the 12 h before the procedure. Nevertheless, this occurred in only 16 patients, with a resulting group of 115 suitable for primary outcome analysis, in agreement with the desired statistical power of the study. Third, different types of local anaesthetic were used for PSGB which varied based on local habits. However, according to previous literature, the extent of effectiveness was similar across different local anaesthetic agents.^17^ Fourth, we do not have information about how implantable devices were programmed or if the programming had been significantly changed after hospital admission. The number of ATPs and shocks delivered is influenced by ICD programming. However, a European survey^40^ showed that in 70% of cases a standard programming including long detection times and ATP before shock was chosen after implantation and that slow VTs were generally only monitored. Fifth, an event adjudication committee was not planned. Data were evaluated and collected by the local investigators in the various centres who were not blinded to treatment and outcome. Sixth, we considered the number of ATPs and shocks (both internal and external) together because we wanted to compute all the treated arrhythmic episodes. This prevents us from being able to differentiate the analyses according to the type of treatment. Seventh, we did not look for changes in the ipsilateral temperature as a marker of acute efficacy. However, it would have been very demanding to provide every centre with the necessary equipment, and probably a change in skin temperature would have been difficult to ascertain in patients suffering from low perfusion or even cardiac arrest. Finally, we did not follow up patients after hospital discharge, so we cannot provide information on potential longer term effects of PSGB.

## Conclusions

This large multicentre prospective observational study outlines PSGB as a highly effective and safe treatment for patients with refractory ES. Whilst data from randomized clinical trials are needed, these findings support its emergency use in life-threatening situations.

## Supplementary Material

ehae021_Supplementary_Data

## Data Availability

Data are available and will be shared after specific and reasonable request to the corresponding author.

## Pre-registered Clinical Trial Number

The pre-registered clinical trial number is NCT05720936.
