# Correction to: Electrical storm treatment by percutaneous stellate ganglion block: the STAR study

**DOI:** 10.1093/eurheartj/ehae631

**Published:** 2024-09-17

This is a correction to: Simone Savastano, Enrico Baldi, Sara Compagnoni, Roberto Rordorf, Antonio Sanzo, Francesca Romana Gentile, Veronica Dusi, Simone Frea, Carol Gravinese, Filippo Maria Cauti, Gianmarco Iannopollo, Francesco De Sensi, Edoardo Gandolfi, Laura Frigerio, Pasquale Crea, Domenico Zagari, Matteo Casula, Giuseppe Sangiorgi, Simone Persampieri, Gabriele Dell’Era, Giuseppe Patti, Claudia Colombo, Giacomo Mugnai, Francesco Notaristefano, Alberto Barengo, Roberta Falcetti, Giovanni Battista Perego, Giuseppe D’Angelo, Nikita Tanese, Alessia Currao, Vito Sgromo, Gaetano Maria De Ferrari, the STAR study group, Electrical storm treatment by percutaneous stellate ganglion block: the STAR study, *European Heart Journal*, Volume 45, Issue 10, 7 March 2024, Pages 823–833, https://doi.org/10.1093/eurheartj/ehae021

In the originally published version of this article, author Giovanni Battista Perego's affiliation was incorrectly listed. This error has been corrected online.

