# Supplementary material for: Electrical storm treatment by percutaneous stellate ganglion block: the STAR study
Source: Eur Heart J. 2024 Jan 30;45(10):823–33. doi: 10.1093/eurheartj/ehae021 (PMC10919918; doi:10.1093/eurheartj/ehae021)
Supplement: ehae021_Supplementary_Data [file ehae021_supplementary_data.zip › eCRF STAR.pdf]

Patient

Record ID	__________________________________
Age	__________________________________
Gender	◯ Male<br>◯ Female
Cardiological History	☐ Hypertensive Cardiopathy<br>☐ Ischemic Cardiopathy<br>☐ Channelopathy<br>☐ Idiopathic Cardiomyopathy<br>☐ Left ventricle dysfunction (LVEF< 50%)
Causative diagnosis of electric storm	◯ STEMI<br>◯ NSTEMI<br>◯ Dilated cardiomyopathy<br>◯ Hypertrophic cardiomyopathy<br>◯ Long QT syndrome<br>◯ Brugada syndrome<br>◯ Catecholaminergic polymorphic ventricular tachycardia (CPVT)<br>◯ Arrhythmogenic right ventricular cardiomyopathy (ARVC) (only right; only left ventricle, biventricular<br>◯ Other
Please, specify the other cause of electric storm	__________________________________
Comorbidities / Risk Factors	☐ Arterial hypertension<br>☐ Diabetes type 1<br>☐ Diabetes type 2<br>☐ Chronic kidney disease<br>☐ Chronic kidney disease with dialytic treatment<br>☐ Vasculopathy<br>☐ Thyroid goitre
Was a cardiac MRI performed during Hospital stay?	◯ No ◯ Yes

PSGB

Pre-PSGB clinical condition

Progressive number of PSGB for the patient

____

Date (DD/MM/YEAR)

____

Hour (24-hour format: HH:MM)

____

Left Ventricle Ejection Fraction (LVEF) %

____
((Please consider the closest LVEF to the PSGB))

Associated morbid condition/s

- ☐ Fever
- ☐ Sepsis without shock
- ☐ Septic shock
- ☐ Thyrotoxicosis
- ☐ Cardiogenic shock
- ☐ Other

Please, specify the other/s morbid condition/s

____

Was VT ablation attempted before PSGB?

- ○ No
- ○ Yes

Was the ablation attempted for the same VT treated with PSGB?

- ○ No
- ○ Yes

Was the attempt effective?

- ○ No
- ○ Yes

((Please consider the non-inducibility of clinical VT at the end of the ablation))

Was the patient intubated before PSGB?

- ○ No
- ○ Yes

Type of sedation/anaesthesia before PSGB

- ○ No, or minimal sedation
- ○ Moderate o deep sedation
- ○ General anaesthesia

Was the patient on antiplatelet therapy at the moment of PSGB?

- ○ No
- ○ Yes, single antiplatelet therapy (SAPT)
- ○ Yes, dual antiplatelet therapy (DAPT)

Was the patient on anticoagulant therapy (OAC/NOAC treatment) at the moment of PSGB?

- ○ No
- ○ Yes, chronic treatment (VKA/DOAC)
- ○ Yes, heparin

Was the patient on ECMO at the moment of PSGB?

- ○ No
- ○ Yes

Was the patient treated with an intra-aortic balloon pump (IABP) at the moment of PSGB?	◯ No<br>◯ Yes
Was the patient under therapeutic hypothermia at the moment of PSGB?	◯ No<br>◯ Yes
Celsius degrees just before PSGB	__________________
Heart rhythm at the moment of PSGB	◯ Ventricular fibrillation (VF)<br>◯ Ventricular tachycardia (VT)<br>◯ Sinus rhythm<br>◯ Other rhythm
Heart rate at the moment of PSGB (bpm)	__________________
Is a 30-minute ECG recording of sinus rhythm available before PSGB?	◯ No<br>◯ Yes
Heart rate in sinus rhythm (bpm)	__________________
Last available plasmatic potassium concentration before PSGB (mEq/L)	__________________<br>((please enter one decimal digit))
Which arrhythmias during electrical storm	◯ Only VT<br>◯ Only VF<br>◯ VT and VF
VT cycle (msec)	__________________<br>(consider the last pre-PSGB VT)
Number of shocks and/or ATP in the hour before PSGB	__________________<br>(for shock consider both internal shocks and external shocks (manual defibrillator or AED))
Number of shocks and/or ATP from 3 to 1 hour before PSGB	__________________<br>(for shock consider both internal shocks and external shocks (manual defibrillator or AED))
Number of shocks and/or ATP from 12 to 3 hours before PSGB	__________________<br>(for shock consider both internal shocks and external shocks (manual defibrillator or AED))
Ongoing oral therapy	☐ amiodarone<br>☐ mexiletine<br>☐ sotalol<br>☐ bisoprolol<br>☐ metoprolol<br>☐ carvedilol<br>☐ propranolol

Ongoing i.v non-antiarrhythmic therapy	☐ dopamine<br>☐ dobutamine<br>☐ epinephrine<br>☐ norepinephrine
Ongoing i.v. antiarrhythmic drugs	☐ amiodarone<br>☐ lidocaine<br>☐ procainamide
Ongoing i.v. beta-blockers	☐ esmolol<br>☐ metoprolol

PSGB characteristics

Tipe of PSGB approach	◯ Anatomical landmark<br>◯ Echo-guided
Side	◯ Left<br>◯ Right
Mode of administration	◯ Bolus<br>◯ Infusion<br>◯ Bolus + infusion
Anaesthetic drug used for PSGB	◯ Lidocaine<br>◯ Bupivacaine<br>◯ Lidocaine + Bupivacaine<br>◯ Other anaesthetic<br>◯ Lidocaine + other anaesthetic
Lidocaine bolus dose (mg)	________________________
Bupivacaine bolus dose (mg)	________________________
Please specify other/s anaesthetic/s used	________________________
other anaesthetic bolus dose (mg)	________________________<br>(mg)
Anaesthetic drug used for infusion	◯ Lidocaine<br>◯ Bupivacaine<br>◯ Other anaesthetic
Please specify other/s anaesthetic/s	________________________
Infusion rate (mg/min)	________________________<br>(in case of changes, please enter the most used infusion rate during drug infusion)
Infusion length (min)	________________________

Arrhythmic relapses during infusion?	◯ No<br>◯ Yes
Kind of relapsed arrhythmias ?	◯ Only sustained VT<br>◯ Only VF<br>◯ VT and VF
Number of shocks/ATP during infusion	__________________________________

PSGB outcome

Anisocoria appearance after PSGB	◯ No<br>◯ Yes<br>((in case of infusion, please consider the beginning of the infusion))
Time between PSGB and the appearance of anisocoria (min)	__________________________________<br>((in case of infusion, please consider the beginning of the infusion))

WARNING: In case of infusion (or bolus + infusion) consider the outcome at the end of infusion, whereas in case of bolus consider the outcome just after the bolus.

Is a 30-minute ECG recording of sinus rhythm available after PSGB?	◯ No<br>◯ Yes
Heart rate in sinus rhythm (bpm)	__________________________________
Arrhythmic relapses after the PSGB?	◯ No<br>◯ Yes
Which arrhythmias during electrical storm	◯ Only VT<br>◯ Only VF<br>◯ VT and VF
VT cycle (msec)	__________________________________<br>(consider the last pre-PSGB VT)
Time between PSGB and first arrhythmic relapse (min)	__________________________________
Number of shocks and/or ATP in the first hour after PSGB	__________________________________<br>(for shock consider both internal shocks and external shocks (manual defibrillator or AED))
Number of shocks and/or ATP from 1 to 3 hours after PSGB	__________________________________<br>(for shock consider both internal shocks and external shocks (manual defibrillator or AED)

Number of shocks and/or ATP from 3 to 12 hours after PSGB	______________________ (for shock consider both internal shocks and external shocks (manual defibrillator or AED))
PSGB-related complications?	◯ No<br>◯ Yes
Kind of complication:	☐ simple hematoma<br>☐ hematoma requiring specific treatment<br>☐ non-complicated vascular injection<br>☐ complicated vascular injection<br>☐ brachial plexus damage<br>☐ simple vascular damage<br>☐ vascular damage requiring specific treatment<br>☐ other
Please, specify the other/s complication/s occurred	______________________

Patient outcome

Was a VT ablation attempted after PSGB?	◯ No<br>◯ Yes
VT ablation was attempted for the same VT treated with PSGB?	◯ No<br>◯ Yes
When was VT ablation attempted?	◯ During index hospitalization<br>◯ In a scheduled hospitalization<br>◯ Unknown
Was VT ablation attempt effective?	◯ No<br>◯ Yes<br>((please consider non-inducibility of clinical VT))
Was cardiac sympathetic denervation performed?	◯ No<br>◯ Yes
When was cardiac sympathetic denervation attempted?	◯ During index hospitalization<br>◯ In a scheduled hospitalization<br>◯ Unknown
Was the patient alive at Hospital discharge?	◯ No<br>◯ Yes
Discharge date (DD/MM/YEAR)	______________________
Neurological outcome at discharge (CPC scale)	◯ Good cerebral performance (CPC 1)<br>◯ Mild cerebral dysfunction (CPC 2)<br>◯ Severe cerebral dysfunction (CPC 3)<br>◯ Coma or vegetative status (CPC 4)<br>◯ Cerebral death (CPC 5)<br>◯ Anaesthesia (CPC A)
Date of death (DD/MM/YEAR)	______________________
Cause of death	◯ Electric storm<br>◯ Other cardiological cause<br>◯ Non- cardiological cause
Specify	______________________
