# Supplementary material for: Electrical storm treatment by percutaneous stellate ganglion block: the STAR study
Source: Eur Heart J. 2024 Jan 30;45(10):823–33. doi: 10.1093/eurheartj/ehae021 (PMC10919918; doi:10.1093/eurheartj/ehae021)
Supplement: ehae021_Supplementary_Data [file ehae021_supplementary_data.zip › Figure 1S.docx]

**Figure 1S**


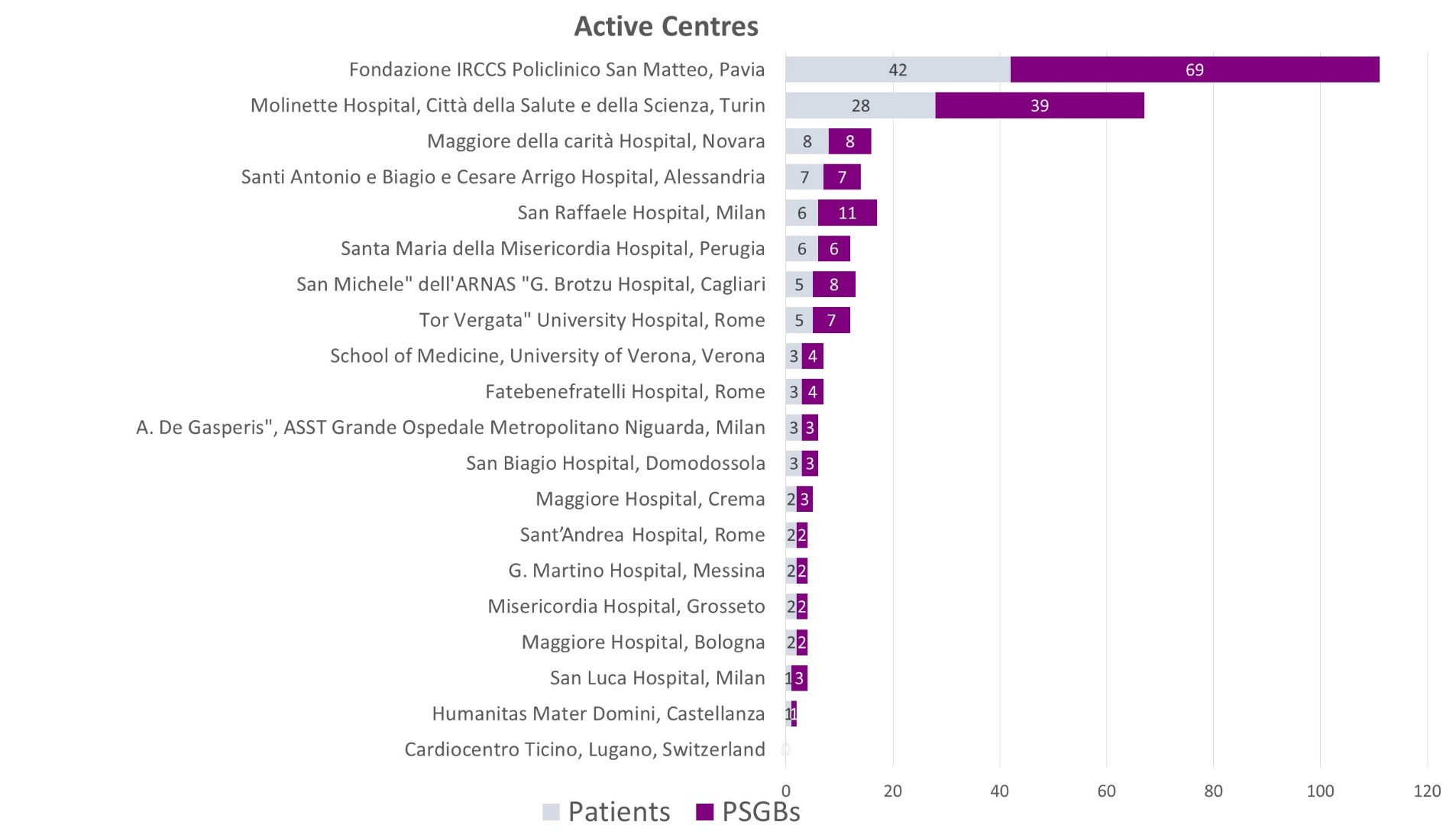


**Figure 1S**: List of the participating centres with the corresponding number of patients enrolled and of procedures performed.
