# Supplementary material for: Electrical storm treatment by percutaneous stellate ganglion block: the STAR study
Source: Eur Heart J. 2024 Jan 30;45(10):823–33. doi: 10.1093/eurheartj/ehae021 (PMC10919918; doi:10.1093/eurheartj/ehae021)
Supplement: ehae021_Supplementary_Data [file ehae021_supplementary_data.zip › Figure 2S.docx]

**Figure 2S**


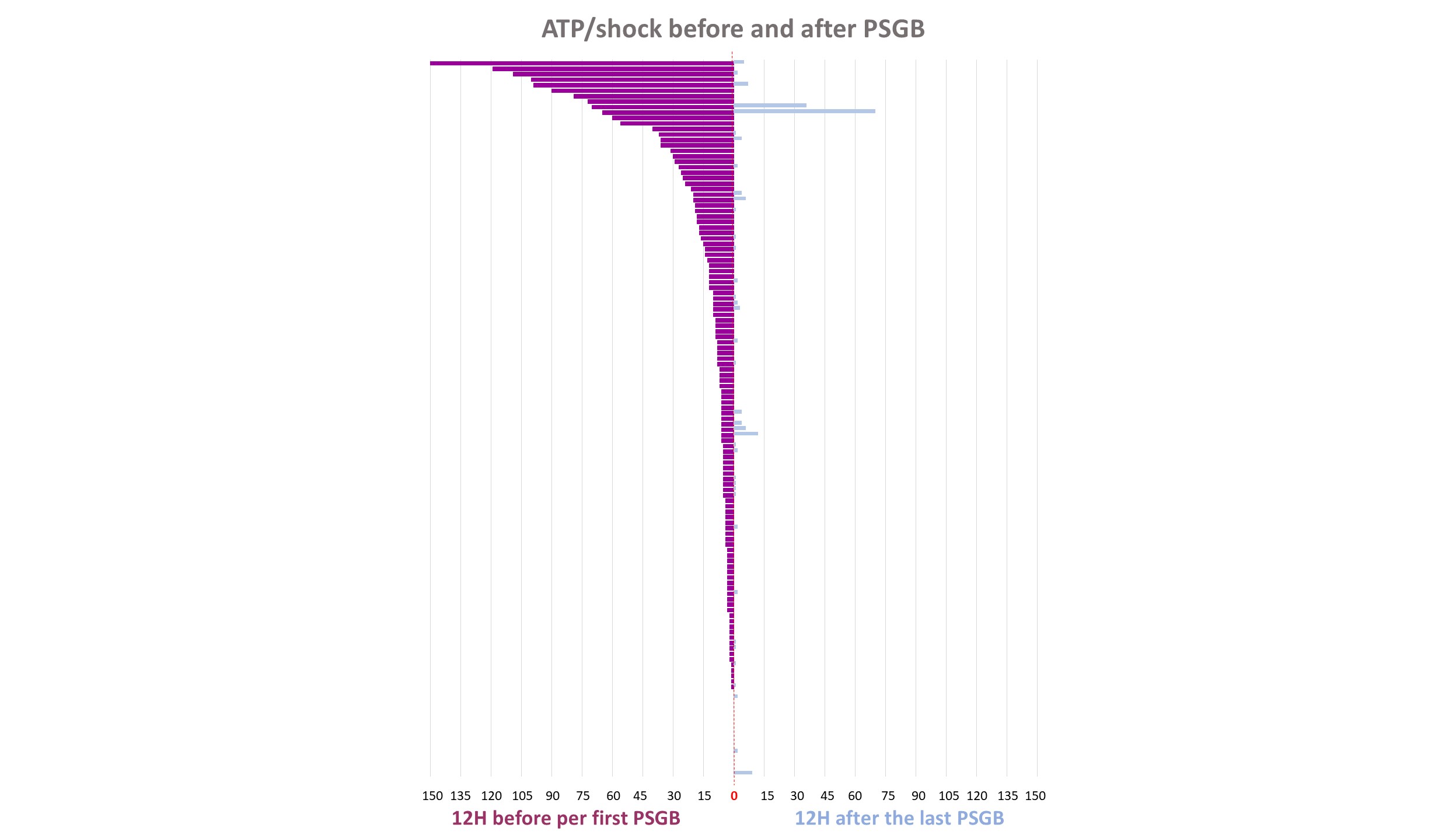


**Figure 2S**: This figure shows for each patient the arrhythmic burden in the 12 hours before the first PSGB and in the 12 hours after the last PSGB.
