# Supplementary material for: Electrical storm treatment by percutaneous stellate ganglion block: the STAR study
Source: Eur Heart J. 2024 Jan 30;45(10):823–33. doi: 10.1093/eurheartj/ehae021 (PMC10919918; doi:10.1093/eurheartj/ehae021)
Supplement: ehae021_Supplementary_Data [file ehae021_supplementary_data.zip › Supplementary Table 1.docx]

**Supplementary Table 1 Training course program.**

| 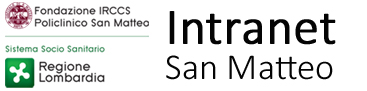**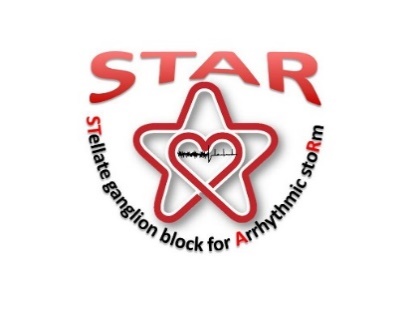**  **PERCUTANEOUS STELLATE GANGLION BLOCK COURSE**  **PROGRAM** | | |
| --- | --- | --- |
| **Timetable** | **Topic** | **Aim of the session** |
| 08:30 – 9:00 | **Partecipants’ registration** |  |
| 09:00 – 10:30 | **Plenary session:**   - Ventricular arrhythmias - Treatment of electrical storm - Rationale, safety and effectiveness of Percutaneous Stellate Ganglion Block | This session provides an overview about ventricular arrhythmias, electrical storm and introduces the rational of PSGB |
| 10:30-11:30 | **Plenary session:**   - Neck anatomy; anatomical and ultrasound-guided approach to the stellate ganglion | This session provided the anatomical basis to approach the stellate ganglion. Here the two approaches are described |
| 11:30-12:30 | **Plenary session:**   - ACLS algorithm and stellate ganglion block in cardiac arrest patients - The STAR study: rational, data collection, procedure, e-CRF | In this session we show when to introduce PSGB in the advanced cardiac life support (ACLS) algorithm for the treatment of patients in cardiac arrest due to refractory VT/VF |
| 12:30- 13:30 | Lunch and coffee |  |
| 13:30- 17:30 | **Hands-on workshops:**   1. Anatomical technique for PSGB and equipment needed 2. Neck ultrasound-anatomy and ultrasound-guided block training with a 3D model 3. ACLS scenario with stellate ganglion block 4. Cases analysis: indications, complications, discussion and brain storming | Three hours of practical activities.  4 stations with groups switch every 45 minutes.  In station #1 students learn how to identify the anatomical landmark on healthy volunteers.  In station #2 students are trained in echo-anatomy of the neck and in ultrasound-guided approach to the stellate ganglion with a 3D model.  In station #3 they deal with simulated cases of VF cardiac arrest and ES.  Station #4 is aimed to clarify any doubt about indications, technique, equipment, possible complications and their management. |
| 17:30-18:00 | Written multiple-choice examination | A 40-question quiz is provided covering the main topics of the course and STAR protocol procedures. |
| 18:00 | **Plenary session:** conclusion and closure of course |  |
