# Supplementary material for: Electrical storm treatment by percutaneous stellate ganglion block: the STAR study
Source: Eur Heart J. 2024 Jan 30;45(10):823–33. doi: 10.1093/eurheartj/ehae021 (PMC10919918; doi:10.1093/eurheartj/ehae021)
Supplement: ehae021_Supplementary_Data [file ehae021_supplementary_data.zip › Supplementary Table 2.docx]

**Supplementary Table 2** Treated arrhythmic events before the procedure, effectiveness of the first, the second or the third PSGB and time passed between the successive procedures.

|  | **Time** | **Progressive number of PSGB per patient** | **Median (IQR) number of ATP/shock 1 hour before PSGB** | **Hodges-Lehmann median differences and 95%CI of the number of ATP/shock 1 hour before and after PSGB** | **p value** |
| --- | --- | --- | --- | --- | --- |
|  |  | **First PSGB** | 2 (0-6.2) | - 2.5 (-3.5 to – 2) | <0.001 |
| Between first and second (min) (IQR) | 790 (425 – 1899) | **Second PSGB** | 2 (7-13) | -2.5 (-3.5 to -0.5) | <0.001 |
| Between second and third (min) (IQR) | 665 (112 – 1528) | **Third or successive** | 1.5 (0-4) | -1.5 (-2 to -0.1) | 0.01 |
|  | | **p value** | n.s | n.s |  |
