# Supplementary material for: Electrical storm treatment by percutaneous stellate ganglion block: the STAR study
Source: Eur Heart J. 2024 Jan 30;45(10):823–33. doi: 10.1093/eurheartj/ehae021 (PMC10919918; doi:10.1093/eurheartj/ehae021)
Supplement: ehae021_Supplementary_Data [file ehae021_supplementary_data.zip › Supplmentary Table 3.docx]

|  | **Approach**  **N= 184** | | |  | |
| --- | --- | --- | --- | --- | --- |
| **Variable** | **Anatomical**  **N=106** | **Ultrasound-guided**  **N=78** | **p value** | |  |
| **Type of centres (%)**  High-volume  Low-volume | 68 (64)  38 (36) | 40 (51)  38 (48) | 0.08 | |  |
| **First attempt (%)** | 75 (71) | 56 (72) | 0.88 | |  |
| **Pre-PSGB refractory cardiac arrest (%)** | 14 (13) | 0 (0) | <0.001 | |  |
| **LVEF (IQR)** | 25 (15-35) | 28 (20-35) | 0.34 | |  |
| **Mode of administration (%)**  Bolus  Bolus and infusion | 91 (86)  15 (14) | 61 (78)  17 (22) | 0.18 | |  |
| **Type of arrhythmias during electrical storm (%)**  VT  VF  VT and VF | 53 (50)  30 (28)  23 (22) | 65 (83)  5 (7)  8 (10) | <0.001 | |  |
| **VT cycle length (IQR) (ms)** | 337 (300-390) | 389 (308-429) | 0.029 | |  |
| **ATP/shock in the hour before (IQR)** | 4 (1-8.2) | 0 (0-3) | <0.001 | |  |
| **Pre-PSGB intervention (%)**  Intubation  Sedation  IABP  ECMO | 29 (27)  16 (15)  11 (10)  6 (6) | 8 (10)  14 (18)  7 (9)  1 (1) | 0.004  0.4  0.7  0.12 | |  |
| **Anti-coagulant/anti-platelet therapy (%)**  None  SAPT only  SAPT + heparin  SAPT + VKA/DOAC  DAPT only  DAPT + heparin  Heparin only  VKA/DOAC only  DAPT + VKA/DOAC | 19 (18)  18 (17)  22 (21)  2 (2)  8 (7)  13 (12)  10 (9)  14 (13)  0 (0) | 7 (9)  17 (22)  7 (9)  4 (5)  5 (6)  3 (4)  14 (18)  20 (26)  1 (1) | 0.09  0.4  0.03  0.09  0.77  0.04  0.08  0.03  0.2 | |  |
| **Post-PSGB anisocoria (%)** | 35 (33) | 37 (47) | <0.001 | |  |
| **PSGB major complications (%)**  Respiratory depression | 0 (0%) | 1 (1.3) | 0.24 | |  |
| **PSGB minor complications (%)**  Bradycardia  Hypotension | 0 (0)  0 (0) | 1 (1.3)  1(1.3) | 0.24  0.24 | |  |
| **PSGB described side effects (%)**  Temporary brachial plexus paralysis  Hoarseness  Dysphonia  Neck pain  Vomit | 0 (0)  0 (0)  0 (0)  1 (0.9)  1 (0.9) | 3 (3.8)  2 (2.5)  1 (1.3)  0 (0)  0 (0) | 0.04  0.09  0.24  0.39  0.39 | |  |

**Supplementary Table 3**: comparison of procedure characteristics comparing anatomical and ultrasound-guided approach
